# Preliminary analysis of double‐negative T, double‐positive T, and natural killer T‐like cells in B‐cell chronic lymphocytic leukemia

**DOI:** 10.1002/cam4.6015

**Published:** 2023-05-04

**Authors:** Luciana Valvano, Filomena Nozza, Giovanni D'Arena, Fiorella D'Auria, Luciana De Luca, Giuseppe Pietrantuono, Giovanna Mansueto, Oreste Villani, Simona D'Agostino, Daniela Lamorte, Giovanni Calice, Teodora Statuto

**Affiliations:** ^1^ Laboratory of Clinical Research and Advanced Diagnostics Centro di Riferimento Oncologico della Basilicata (IRCCS‐CROB) Rionero in Vulture Italy; ^2^ Immunohematology and transfusional medicine "S. Luca" Hospital, ASL Salerno Vallo della Lucania Italy; ^3^ Laboratory of Clinical Pathology Centro di Riferimento Oncologico della Basilicata (IRCCS‐CROB) Rionero in Vulture Italy; ^4^ Hematology and Stem Cell Transplantation Unit Centro di Riferimento Oncologico della Basilicata (IRCCS‐CROB) Rionero in Vulture Italy; ^5^ Laboratory of Preclinical and Translational Research Centro di Riferimento Oncologico della Basilicata (IRCCS‐CROB) Rionero in Vulture Italy

**Keywords:** chronic lymphocytic leukemia, double‐negative T cells, double‐positive T cells, flow cytometry, immune surveillance, lymphocytes, natural killer T‐like cells

## Abstract

**Background:**

B‐cell chronic lymphocytic leukemia (B‐CLL) is characterized by the expansion of CD5^+^ malignant B lymphocytes. Recent discoveries have shown that double‐negative T (DNT) cells, double‐positive T (DPT) cells, and natural killer T (NKT)‐cells may be involved in tumor surveillance.

**Methods:**

A detailed immunophenotypic analysis of the peripheral blood T‐cell compartment of 50 patients with B‐CLL (classified in three prognostic groups) and 38 healthy donors (as controls) matched for age was performed. The samples were analyzed by flow cytometry using a stain‐lyse‐no wash technique and a comprehensive six‐color antibody panels.

**Results:**

Our data confirmed a reduction in percentage values and an increase in absolute values of T lymphocytes in patients with B‐CLL, as already reported. In particular, DNT, DPT, and NKT‐like percentages were significantly lower than in the controls, except for NKT‐like in the low‐risk prognostic group. Moreover, a significant rise in the absolute counts of DNT cells in each prognostic group and in the low‐risk prognostic group of NKT‐like cells was found. A significant correlation of the absolute values of NKT‐like cells in the intermediate‐risk prognostic group versus B cells was observed. Furthermore, we analyzed whether the increase in T cells was related to the subpopulations of interest. Only DNT cells were positively correlated with the increase in CD3^+^ T lymphocytes, regardless of the stage of the disease, supporting the hypothesis that this T‐cell subset plays a key role in the immune T response in B‐CLL.

**Conclusion:**

These early results supported that DNT, DPT, and NKT‐like subsets may be related to disease progression and should encourage further studies aimed at identifying the potential immune surveillance role of these minority T subpopulations.

## 1 INTRODUCTION

A growing interest in minor T‐cell subsets such as “double‐negative T” (DNT), “double‐positive T” (DPT), and “natural killer T” (NKT) cells has been shown in recent decades, due to their relevant role in tumors. The migration of DNT (CD3^+^ CD4^−^ CD8^−^) cells from bone marrow to thymus marks the beginning of the process of T‐cell development. In the thymic cortex, DNT cells gradually differentiate and in the last stage of development DNT are converted into DPT (CD3^+^ CD4^+^ CD8^+^) cells. Finally, the mature single positive T cells (CD3^+^ CD4^+^ or CD3^+^ CD8^+^) pass from medulla to peripheral circulation.^1^, ^2^

A minor subset of mature (post‐thymic) DNT cells is normally found in circulating blood of healthy individuals (usually 1%–5% of peripheral lymphocytes).^3^ DNT cells are divided into two groups, αβ + DNT and γδ + DNT, according to different T‐cell receptors (TCRs). It has been suggested that DNT cells can secrete a vast arrangement of cytokines and have a double effect by eliminating tumor cells and impeding graft‐versus‐host disease, as recently showed by experimental works.^3^, ^4^, ^5^, ^6^, ^7^

Furthermore, DPT cells are differentiated on the basis of the intensity of their expression marker (CD4^high^CD8^low^ and CD4^low^CD8^high^).^8^, ^9^, ^10^ The presence of DPT in the peripheral blood (PB) (2%–3%)^8^ of healthy individuals has been ascribed to the early release of CD4^+^ CD8^+^ T cells from the thymus to periphery^11^, ^12^ or from the peripheral acquisition of the second positive marker (CD4 or CD8).^13^ Finally, it has been demonstrated that DPT cells exhibit cytotoxic potential or a regulatory role in various tumors.^12^

NKT are T cells with NK cell‐like phenotype and function. They are classified as two different populations on the basis of TCR: “invariant natural killer T” (iNKT) and “non invariant” NKT cells.^14^ They represent about 1% of all PB T‐cells.^15^ In humans, iNKT cells express a semi‐invariant TCR containing the Vα24‐Jα18 chain and react with lipid antigens presented by the HLA class I–related molecule CD1d, releasing regulatory cytokines and costimulatory molecules, showing their capacity to repress or activate immune responses, including immune surveillance against tumors. In cancer patients, decreased iNKT cell numbers correlate with adverse prognosis.^16^, ^17^, ^18^, ^19^, ^20^, ^21^ The “non invariant” NKT cells have a different TCR repertoire and can suppress anti‐tumor responses.^17^, ^18^, ^19^, ^20^, ^21^, ^22^

A diagram depicting the origin and previously observed roles of DNT, DPT, and NKT cells is given in Figure S1.

A significant increase in total T lymphocytes has previously been reported in patients with B‐cell chronic lymphocytic leukemia (B‐CLL).^23^, ^24^ B‐CLL is a common adult neoplasm in which monomorphic small mature CD5^+^, CD23^+^, CD200^+^ B cells account for more than 5 × 10^9^/L in PB.^25^, ^26^, ^27^, ^28^, ^29^ In most cases, subjects with B‐CLL displayed only a clonal lymphocytosis in their blood. Less often, lymphadenopathy, splenomegaly, anemia, or thrombocytopenia can lead to the diagnosis.

Given the retrospective nature of the study and the inability to assess CD1d and/or V‐alpha‐24 receptor expression, we rather identified an NKT‐like subpopulation that is phenotypically characterized by coexpression of TCR with NK‐associated receptors, such as CD56 or CD16.^30^

Due to the increasing interest in the study of these minor T subpopulations and their potential roles in tumors, including as a novel adoptive cell therapy for cancer, we investigated DNT, DPT, and NKT‐like values by flow cytometry in 50 B‐CLL patients and on 38 healthy donors, as controls.

## 2 MATERIALS AND METHODS

### 2.1 Patients

Patients with untreated B‐CLL and admitted at our institution from December 2008 to November 2021 were identified in this retrospective study. Patients were classified in three prognostic groups, as 0/A or 1/A (low‐risk), 1/B and 2/B (intermediate‐risk), and 3/C and 4/C (high‐risk), according to the Rai and Binet staging systems (26). Percentage and absolute (/μL) values of NKT‐like (CD3^+^ CD16^+^ CD56^+^), DNT (CD3^+^ CD8^−^ CD4^−^ CD56^−^) and DPT (CD3^+^ CD8^+^ CD4^+^) cells were evaluated by flow cytometry in 50 PB of patients with B‐CLL and 38 healthy donors. Main clinical–biological features of patients are reported in Table 1. All study participants were over 18 years of age and all had undergone flow cytometric analysis for lymphocyte typing (B, T, and NK cells). This noninterventional study was conducted according to the principles of the Declaration of Helsinki and approved by Comitato Etico Unico Regionale per la Basilicata (approval number 20200026759). Written informed consents were obtained from the participants for the publication of any potentially identifiable data included in this article.

**TABLE 1:** Clinical–biological features of patients with B‐CLL and healthy donors.

Clinical–biological features		Age, median (range)^1^	Males, *N* (%)^1^	Splenomegaly, *N* (%)^1^	Adenopathy, *N* (%)^1^	IgVH unmutated, *N* (%)^2^	CD38‐positive expression, *N* (%)^3^	WBC count (μL^−1^), median (IQR)^1^	Ly count (μL^−1^), median (IQR)^1^	Hb levels (g/dL), median (IQR)^1^	PLT count (×10^9^/L), median (IQR)^1^	Treated patients, *N* (%)^1^ Treatment Time to treatment (months) Response	FISH, *N* (%)^4^
*N* patients: 50		70.5 (49–89)	33 (66)	16 (32)	22 (44)	15 (48.4)	3 (27.3)	20,675 (14,360)***	13,800 (13,620)***	13.3 (9)	165 (71)	9 (18)^1^	
Healthy donors: 38		66.5 (46–84)	22 (58)					5490 (2310)	1700 (850)	13.80 (28)	197 (87.5)		
Staging	Rai–Binet stage *N* (%)^1^												
Group A (low risk)	0/A 21 (42)	73 (50–85)	11 (22)	0 (0)	0 (0)	2 (6.5)	1 (9.1)	15,720 (11415)^n.a.^	10,400 (7425)^n.a.^	14.4 (10.5)^n.a.^	178 (87.5)^n.a.^	1 (2), Bendamustine, 27, PR	7 (18.9)^a^ 3 (8.1)^b^ 1 (2.7)^c^ 2 (5.4)^b,c^
	1/A 3 (6)	73 (65–89)	2 (4)	0 (0)	2 (4)	1 (3.2)	0 (0)	11,580 (212,340)^n.a.^	5100 (16,200)^n.a.^	15.4 (9)^n.a.^	207 (104)^n.a.^		1 (2.7)^c^
Total	24 (48)	73 (59–89)	13 (26)	0 (0)	2 (4)	3 (9.7)	1 (9.1)	15,630 (11,935)***	10,200 (7625)***	14.5 (10)^n.a.^	178.5 (75.5)		
Group B (intermediate risk)	1/B 6 (12)	70 (55–83)	5 (10)	4 (8)	2 (4)	2 (6.5)	0 (0)	26,645 (16,740)^n.a.^	19,950 (14,800)^n.a.^	14 (12)^n.a.^	176 (51)^n.a.^		5 (13.5)^a^ 1 (2.7)^c^
	2/B 13 (26)	68 (49–87)	8 (16)	6 (12)	11 (22)	5 (16.1)	1 (9.1)	24,690 (12,885)^n.a.^	16,860 (14,645)^n.a.^	13 (6)^n.a.^	145 (46.5)^n.a.^	1 (2%), Ibrutinib, 6, NR 1 (2%), Bendamustine, 3, PR 1 (2%), FCR, 6, CR	3 (8.1)^a^ 4 (10.8) ^b^ 1 (2.7)^c^ 1 (2.7)^d^ 1 (2.7)^b,d^ 1 (2.7)^c,d^
Total	19 (38)	69 (49–87)	13 (26)	10 (20)	13 (26)	7 (22.6)	1 (9.1)	25,000 (14,080)***	18,600 (16,700)***	13 (8)	154 (49)		
Group C (high risk)	4/C 7 (14)	69 (52–87)	7 (14)	6 (12)	7 (14)	5 (16.1)	1 (9.1)	31,160 (71,900)***	25,600 (74,400)***	11.6 (6)	86 (119)*	1 (2%), Bendamustine, 1, PR 1 (2%) Bendamustine, 3, CR 1 (2%), FCR, 1, CR 1 (2%), FCR, 5, CR 1 (2%), Clorambucile, 36, PR	3 (8.1)^b^ 1 (2.7)^e^ 1 (2.7) ^b,d^ 1 (2.7) ^b,e^

*Note*: Median and interquartile range (IQR) of absolute counts are presented. *p*‐value (evaluated by using Wilcoxon rank analysis): *significantly different from controls, *p* < 0.05; **significantly different from controls, *p* < 0.01; ***significantly different from controls, *p* < 0.001. ^1^Data available for 50 patients; ^2^data available for 31 patients, ^3^data available for 11 patients; ^4^data available for 37 patients; ^a^negative, ^b^del13q, ^c^tris12, ^d^del17p, ^e^del11q.

Abbreviations: CR, complete response; FCR, fludarabine, cyclophosphamide, rituximab; FISH, fluorescent in situ hybridization; Hb, hemoglobin; IgVH, immunoglobulin heavy chain variable region; IQR, interquartile range; Ly, lymphocyte; n.a., not assigned; NR, no response; PLT, platelet; PR, partial response; WBC, white blood count.

### 2.2 Flow cytometry

**FIGURE 1:**
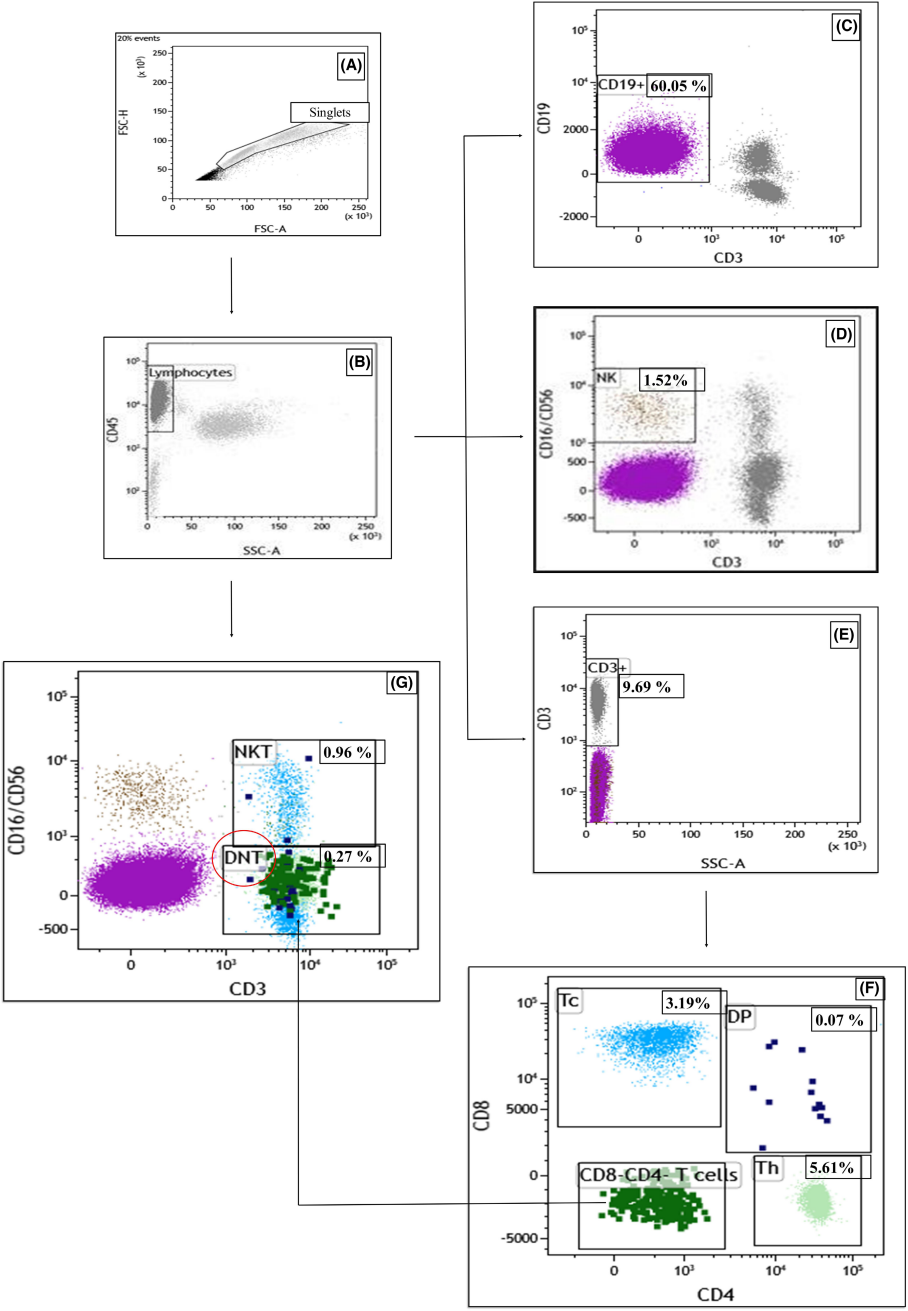
Gating strategy. In our assays, any debris, dead cells and clumps or doublets were excluded using FSC‐Height (FSC‐H) by FSC‐Area (FSC‐A) parameters (A, gate “singlets”). CD45^+^ lymphocytes were gated on CD45 versus SSC dot plot on “singlets” (B). From the lymphocyte gate we sequentially identified B cells (CD19^+^) (C), CD3^−^ CD16^+^ CD56^+^ cells (NK) (D) and total T (CD3^+^) cells (E). From total T cells gate we identified the CD8^+^ CD3^+^ cytotoxic T cells (Tc), the CD4^+^ CD3^+^ T helper cells (Th), the CD8^+^ CD4^+^ CD3^+^ double positive T cells (DP) and the CD8^−^ CD4^−^ CD3^+^ T cells (F). The DNT was obtained by gating the CD8^−^ CD4^−^ CD3^+^ T cells on CD16/CD56^−^, while the NKT‐like cells were obtained from lymphocytes by gating on CD3^+^ and CD16/CD56^+^ (panel G). Median percentages are evaluated on CD45^+^ cells.

PB samples were analyzed at diagnosis by flow cytometry using a stain‐lyse‐no wash technique and a comprehensive six‐color antibody‐panel (FITC/PE/PerCP‐Cy5‐5/PE‐Cy7/APC/APC‐H7 fluorescent conjugates) on a BD FACSCanto™ II (BD Biosciences, BD) or “Navios 10/3” (Beckman Coulter, BC). Lymphocyte subpopulations panels included monoclonal antibodies against these surface antigens: CD3, CD16, CD56, CD45, CD19, CD8, and CD4. The expression of CD38 on malignant cells (CD19^+^ CD5^+^) was also evaluated in only 11 patients. A detailed list of panels used for flow cytometric analysis, including the antibody clone, is reported in Table S1. The pathological B cells were characterized by flow cytometry in other panels (data not shown), also containing CD5. The monoclonal antibodies were purchased from BD or BC. Samples were analyzed by Kaluza Software Version 2.1 (BC): “rare event” display function was used favoring the visualization of small subpopulations. For recognition of the smaller subpopulations, cells clustered by specific markers were identified, providing a sufficient denominator of relevant events in the lymphocyte gate (about 15,000 events). In our assays, any debris, dead cells, and clumps or doublets were excluded using FSC‐Height (FSC‐H) by FSC‐Area (FSC‐A) parameters (Figure 1A, gate “singlets”). CD45^+^ lymphocytes were gated on CD45 versus SSC dot plot on “singlets” (Figure 1B). From the lymphocyte gate, B cells (CD19^+^) (Figure 1C), CD3^−^ CD16^+^ CD56^+^ cells (NK) (Figure 1D) and total T (CD3^+^) cells (Figure 1E) were sequentially identified. CD38 was considered positive when it was at least 20% expressed on CD19^+^ B cells. From the total T‐cell gate, CD8^+^ CD3^+^ cytotoxic T cells (Tc), CD4^+^ CD3^+^ T helper cells (Th), CD8^+^ CD4^+^ CD3^+^ DPT, and CD8^−^ CD4^−^ CD3^+^ T cells were identified (Figure 1F). The DNT was obtained by gating the CD8^−^ CD4^−^ CD3^+^ T cells on CD16/CD56^−^, while the NKT‐like cells were obtained from lymphocytes by gating on CD3^+^ and CD16/CD56^+^ (Figure 1G). To determine the number of these cells per microliter of blood, a double platform was used: the percentage of lymphocytes with immunophenotypic features of DNT, DPT, and NKT‐like cells was multiplied by the number of white blood cells (WBC) per microliter as simultaneously determined by an automated full blood count on the same sample. A general picture of the gates analyzed and the differences highlighted between B‐CLLs and healthy donors is shown in Figure S2.

### 2.3 Statistics

Most of the data series were found to deviate from a normal distribution (by using Shapiro–Wilk test), so possible differences between groups were evaluated by nonparametric statistics. Continuous variables were reported as median and interquartile range (IQR). The differences between groups were investigated by the two‐sided Wilcoxon rank sum test and the Kruskal–Wallis test. The Spearman test was used to evaluate possible correlations of the data series. *p* < 0.05 was considered statistically significant. All analyses were performed by R software^31^ and customized plots were obtained by ggpubr package.^32^

## 3 RESULTS

### 3.1 Percentages of DNT, DPT, and NKT‐like cells in CLL patients and healthy controls

To evaluate the possible presence of differences between the various lymphocyte subpopulations of CLL patients compared to healthy donors, the percentages of the different subpopulations listed here were evaluated: B cells, T cells, NK cells, Tc cells, Th cells, DNT cells, DPT cells, and NKT‐like cells. Comparing each lymphocyte subpopulation between CLL patients and healthy donors, a significant reduction in all T subsets was found (Table S2). In particular, DNT cells have a median value on PB CD45^+^ cells of 0.50% in healthy donors and of 0.27% in B‐CLL patients; DPT cells have a median value on PB CD45^+^ cells of 0.18% in healthy donors and of 0.07% in B‐CLL patients; NKT‐like cells have a median value on PB CD45^+^ cell value of 2.06% in healthy donors and of 0.96% in B‐CLL patients (Figure S3). Furthermore, we compared all the lymphocyte subpopulations of each class of patients stratified by the Rai–Binet staging system (group A: low risk; group B: intermediate risk; group C: high risk) with the healthy donors. Again, the percentages of all lymphocyte subpopulations were significantly lower in CLL prognostic group than in the controls except for group A of NKT‐like cells (Table S2; Figure S3).

### 3.2 Absolute values of DNT, DPT, and NKT‐like cells in CLL patients and healthy donors

Considering the significant differences found in the percentage between the various lymphocyte subpopulations of CLL patients versus healthy controls (Table S2; Figure S3), the subsets by absolute count were evaluated. Surprisingly, the absolute values of the lymphocyte subpopulations analyzed by flow cytometry were all increased in patients with B‐CLL with respect to controls; the increase was not statistically significant only for NK and DPT cells (*p* > 0.05) (Table 2).

**TABLE 2:** Distribution of lymphocyte subset absolute values in the total B‐CLL patients, in the different Rai–Binet stages and in the controls.

Values	B cells	T cells	NK cells	Tc cells	Th cells	DNT cells	DPT cells	NKT‐like cells
Control *n*= 38 Median μL^−1^ (IQR)	158.9 (184.8)	1306 (767.2)	241.3 (210.2)	410.8 (342.7)	1108.2 (620.9)	30.2 (24.2)	10.2 (34.5)	97.2 (144.8)
Total B‐CLL n = 50 Median μL^−1^ (IQR)	13857.6 (14458.1)***	2302 (1515.4)***	333.8 (336.1)	719.4 (686)***	1427.5 (919.3)*	55.1 (83.8)***	18.90 (43)	233.5 (210.1)***
Group A *n* = 24 Median μL^−1^ (IQR)	8230.4 (12434.3)***	2319.7 (1024.8)***	295.3 (408.5)	670.4 (559.2)**	1569.3 (685.5)**	48.6 (75.8)**	15.6 (40.4)	245.5 (195.8)***
Group B *n* = 19 Median μL^−1^ (IQR)	15107.2 (9534.6)***	2049.1 (2312.4)**	349.6 (343.5)	756.3 (1269.4)**	1016.1 (1088.1)	57.3 (83.8)**	19.2 (52.35)	236.7 (264.2)
Group C *n* = 7 Median μL^−1^ (IQR)	24761.8 (64958.3)***	1876.2 (3089.9)	363.9 (886.7)	645.3 (940.7)*	1277.9 (1822.8)	77.2 (95.8)**	25.6 (36.3)	147.1 (185.6)

*Note*: Median and interquartile range (IQR) of absolute (μL^−1^) counts on total leukocytes (CD45^+^) are presented. Asterisks indicate the result of the comparison between the absolute values of the various lymphocyte populations between CLL patients and healthy subjects. *p*‐Value (evaluated by using Wilcoxon rank analysis): *significantly different from controls, *p* < 0.05; **significantly different from controls, *p* < 0.01; ***significantly different from controls, *p* < 0.001.

**FIGURE 2:**
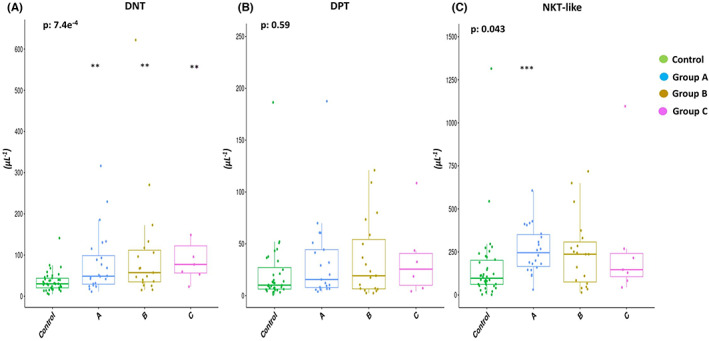
The impact of Rai–Binet staging in the absolute counts of DNT (panel A), DPT (panel B) and NKT‐like (panel C) cells respect to healthy controls. Each symbol represents single subject and vertical bars represent median values. Statistically significant analyses (respect the healthy donors) are indicated by asterisks: *p < 0.05, **p < 0.01 and ***p < 0.001. *p*‐value reported in the box plots panel refers to Kruskal‐Wallis test.

In particular, the impact of Rai–Binet clinical staging was evaluated and, with respect to control, a significant rise in the absolute counts of DNT cells in each prognostic group was found (Group A *p* = 0.0036, Group B *p* = 0.00305, and Group C *p* = 0.00174) (Figure 2A) and in Group A of NKT‐like cells (*p* = 0.000167) (Figure 2C). No significant increase in NKT‐like cells was shown in Group B or C (*p* > 0.05) (Figure 2C) and for DPT cells in any prognostic group (Figure 2B).

In order to identify a possible correlation between the subpopulations of interest, the Spearman pairwise test was performed and no significant difference in absolute values of these subsets was found (either on total patients or by prognostic group, *p* > 0.05).

Moreover, Kruskal–Wallis test showed significant difference between all prognostic groups and healthy donor group only for DNT and NKT cells (*p*‐value is reported in the box plots panel of Figure 2).

### 3.3 Absolute values of WBC, Ly, Hb, and PLT values in CLL patients and healthy donors

To evaluate the impact of the Rai–Binet staging of CLL patients, the absolute count of the following parameters was compared: WBC, lymphocytes (Ly), hemoglobin (Hb), and platelets (PLT) against the controls. Wilcoxon test showed a significant increase in WBCs and Ly in each prognostic groups of patients compared to healthy donors (Table 1; Figure S4A,B). Conversely, there was no significant variation for Hb and PLT values (Table 1; Figure S4C,D), except for Group C of PLT values, which showed a significant reduction (*p* = 0.02) (Figure S4D). Moreover, Kruskal–Wallis test showed significant difference between all prognostic groups and the healthy donor group for all clinical values (*p*‐value is reported in the box plots panel of Figure S4).

To explain a possible antitumor activity/response, the Spearman correlation test of DNT or DPT or NKT‐like cells to B‐cells was performed (the median percentage of CD5 positive on CD19 cells was 99.12, IQR = 1915). Only the absolute values of NKT‐like cells and B cells in prognostic Group B showed a significant correlation (*p* = 0.008862).

### 3.4 Relationship between DNT, DPT, and NKT‐like cells and prognostic factors

In order to identify a possible correlation between B‐CLL prognostic factors (CD38 expression and IgVH mutation) and absolute count of DNT, DPT, and NKT‐like cells, a Wilcoxon rank analysis was performed. These comparisons were conducted on 11 patients for CD38 expression and on 31 patients for IgVH mutation. In three patients (27.3%) CD38 expression was found in more than 20% of leukemic cells (median 62%, IQR: 54%), and eight patients (72.7%) were negative for CD38 expression (median 0.8%, IQR 2.4%).

Comparing the absolute counts of DNT, DPT, and NKT‐like cells and the expression of CD38, we did not find a significant difference in the absolute values of the individual T subpopulations between patients who expressed or did not express CD38 (*p* > 0.05).

The same nonsignificant result was found by analyzing the absolute values of the single T subsets in relation to the IgVH mutation (*p* > 0.05). In particular, 16 patients (51.6%) were IgVH mutated.

### 3.5 Relationship between DNT, DPT, and NKT‐like cells and clinical/personal features

**FIGURE 3:**
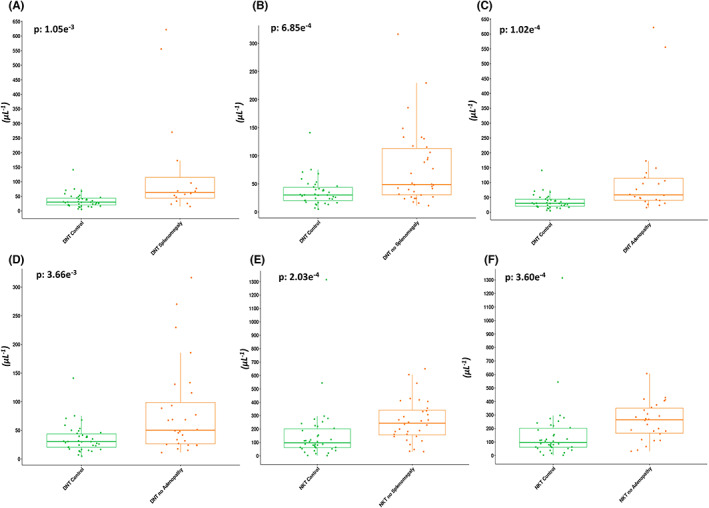
Relationship between DNT or NKT‐like cells and pathological conditions (splenomegaly or adenopathy). Wilcoxon rank test showed significant differences between DNT values (A‐D) with or without pathological conditions (splenomegaly or adenopathy) or NKT‐like values (E,F) only without pathological conditions (splenomegaly or adenopathy) in B‐CLL patients respect the healthy controls. Each symbol represents single subject and vertical bars represent median values. p‐value reported in the box plots panel refers to Wilcoxon test.

To identify the presence of a relationship between DNT, DPT, and NKT‐like cells and clinical/personal features in CLL patients, the absolute counts of each subpopulation stratified by the presence or absence of splenomegaly or adenopathy were compared. Wilcoxon test highlighted no significant differences in the absolute counts of DNT, DPT, and NKT‐like cells between patients with and without splenomegaly or between patients with and without adenopathies (*p* > 0.05) (data not shown). However, with respect to healthy donors, there was a significant increase of DNT absolute values in patients with or without splenomegaly (*p* < 0.01, Figure 3A,B) and in patients with or without adenopathies (*p* < 0.01, Figure 3C,D).

On the contrary, the comparison of absolute values between patients of NKT‐like versus healthy donors showed a significant increase only in patients without splenomegaly/adenopathies (*p* < 0.001, Figure 3E,F). There was no significant difference in absolute DPT counts of healthy donors versus patients with or without splenomegaly/adenopathies (*p* > 0.05) (data not shown). The clinical outcome (as progression‐free survival, PFS) of B‐CLL patients with absolute values of DNT, DPT, and NKT‐like cells was analyzed and we found no significant data (*p* > 0.05). There was no significant difference also between absolute counts of DNT, DPT, and NKT‐like cells and gender both in B‐CLL patients and in controls (*p* > 0.05).

### 3.6 Relationship between DNT, DPT, and NKT‐like cells and the increase in CD3 cells

The increase of T‐cell populations in B‐CLL patients was correlated with the different T‐cell subsets. Spearman's correlation showed that the absolute increase in CD3^+^ lymphocytespositively correlated with each T‐cell subset in all B‐CLL patients (*p* < 0.01, Figure S5). Even in healthy donors, the increase was positively correlated with the T‐cell subsets analyzed (*p* < 0.01, Figure S6).

**FIGURE 4:**
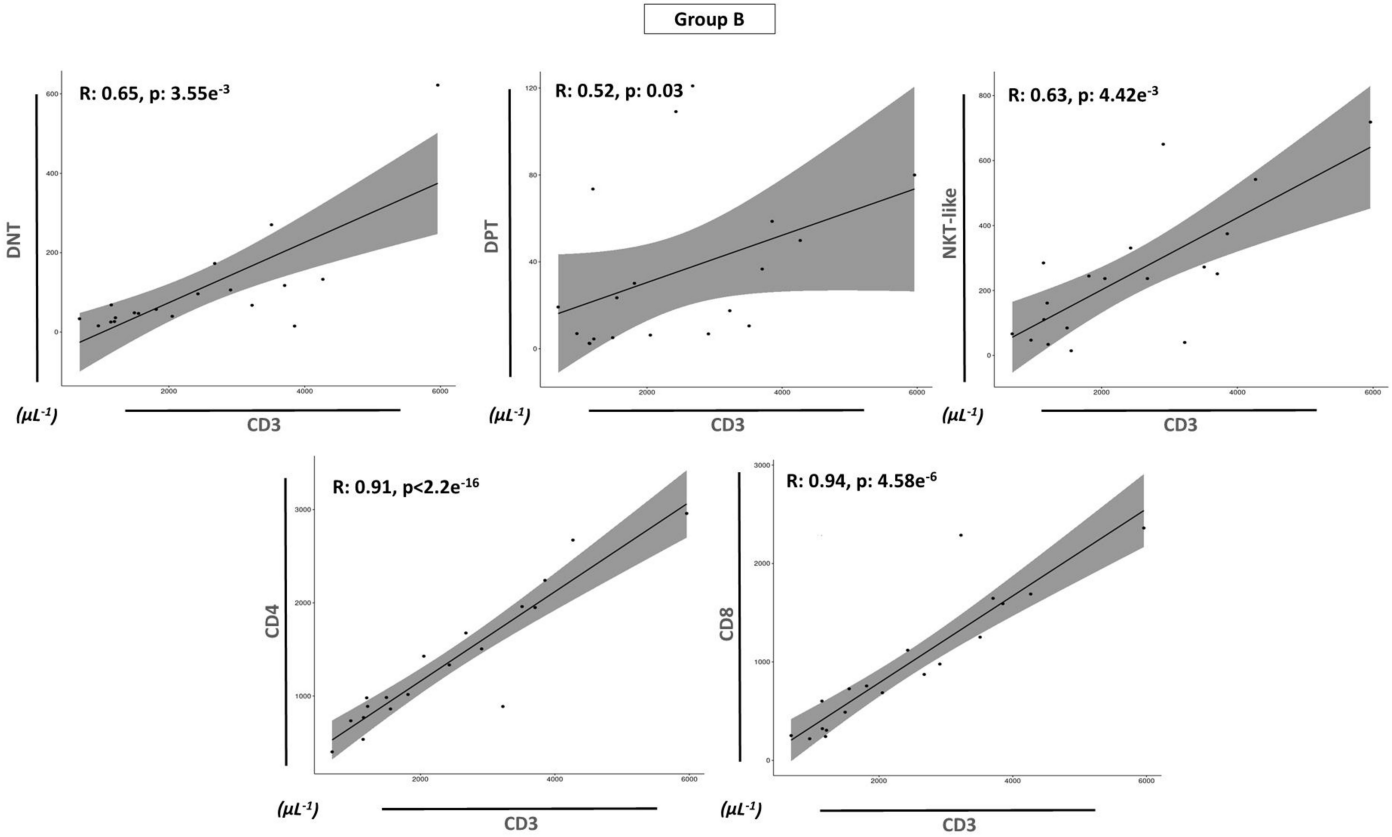
Relationship between each T subsets and the increase of CD3 cells in Group B. Spearman's correlation coefficient (R) analysis between the absolute values of CD3^+^ cells (μL^−1^) and all T‐cell subsets (μL^−1^) (DNT, DPT, NKT, CD8, and CD4) for Group B. Statistical analysis is indicated with the *p*‐value.

**FIGURE 5:**
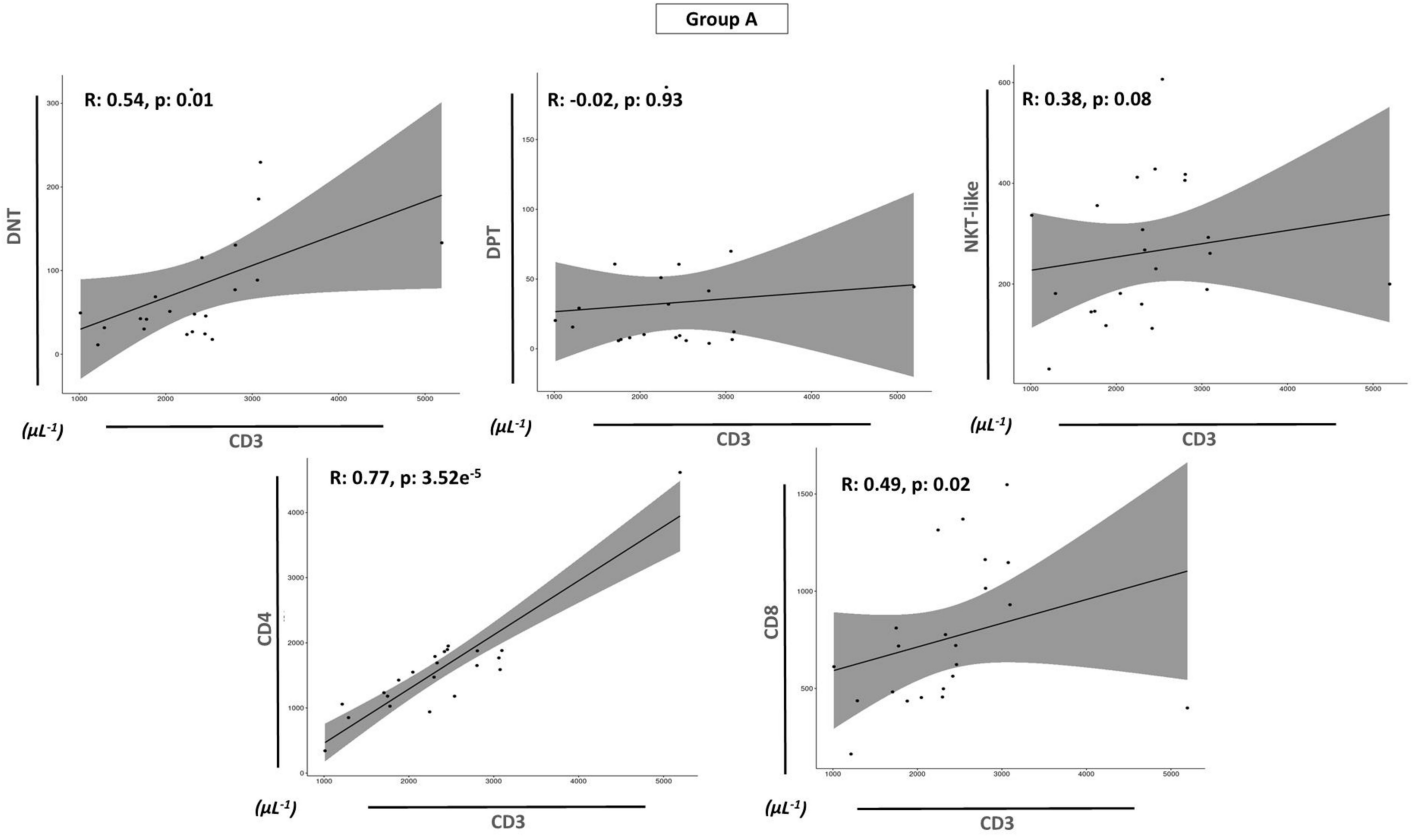
Relationship between each T subsets and the increase of CD3 cells in Group A. Spearman's correlation coefficient (R) analysis between the absolute values of CD3^+^ cells (μL^−1^) and all T‐cell subsets (μL^−1^) (DNT, DPT, NKT, CD8, and CD4) for Group A. Statistical analysis is indicated with the *p*‐value.

**FIGURE 6:**
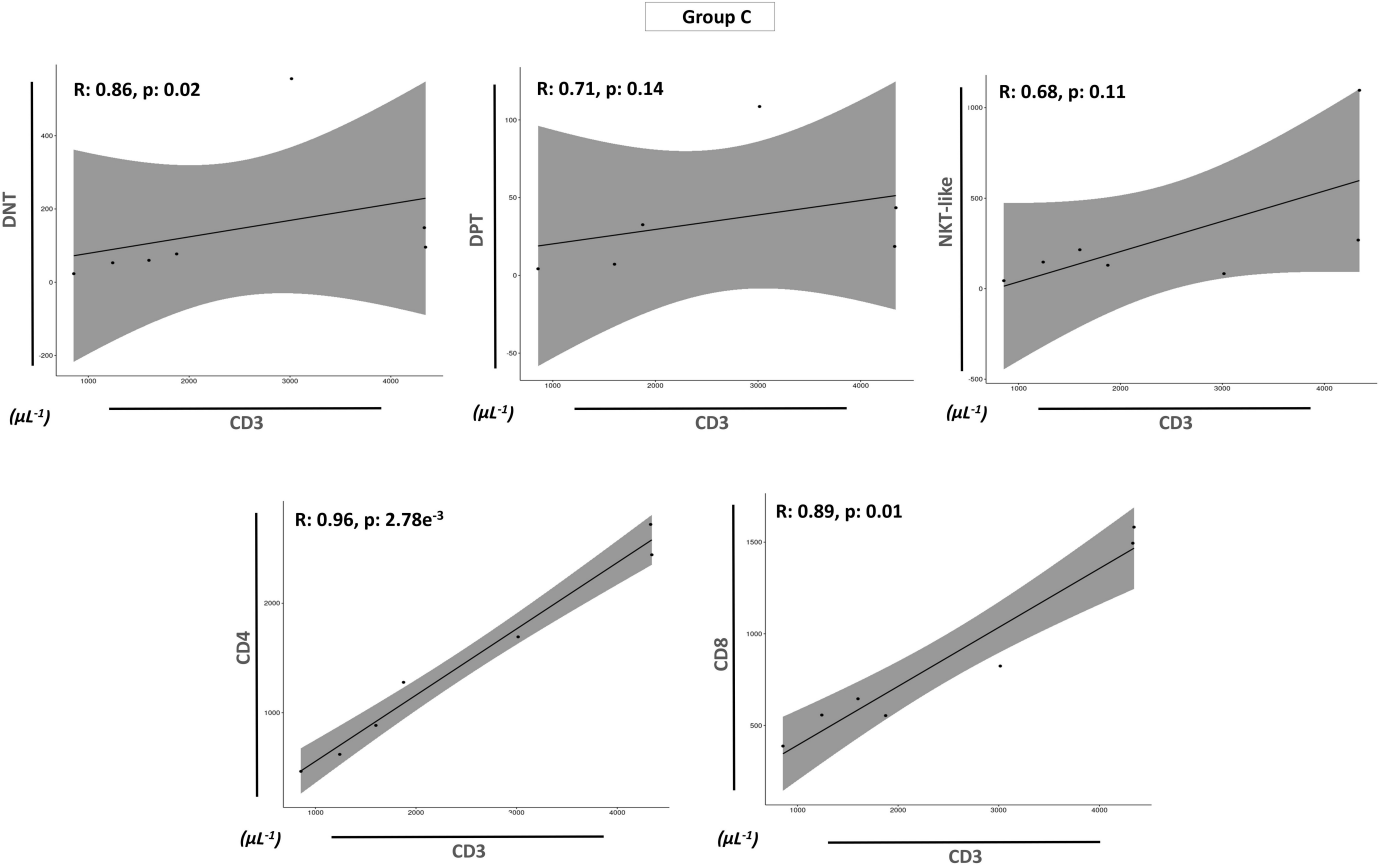
Relationship between each T subsets and the increase of CD3 cells in Group C. Spearman's correlation coefficient (r) analysis between the absolute values of CD3^+^ cells (μL^−1^) and all T‐cell subsets (μL^−1^) (DNT, DPT, NKT, CD8, and CD4) for Group C. Statistical analysis is indicated with the *p*‐value.

Finally, we investigated the correlations between the increased absolute values of CD3^+^ cells with all the T‐cell subsets in all prognostic groups (Groups A, B, and C). An interesting different trend was found: the absolute counts of CD3^+^ cells of Group B showed a positive correlation with the absolute values of all T‐cell subsets (*p* < 0.05, Figure 4), whereas no correlation was found in Group A (Figure 5) and Group C (Figure 6) for DPT and NKT‐like values (*p* > 0.05). In summary, only the DNT (*p* < 0.05), CD8 (*p* < 0.05) and CD4 (*p* < 0.01) cell numbers of each group were significantly and positively correlated in each prognostic group (Figures 4, 5, 6).

## 4 DISCUSSION

In this study, we aimed to analyze variations in absolute counts of lymphocyte subpopulations in patients with B‐CLL with respect to healthy donors. To this end, eight different subpopulations of T, B, and NK lymphocytes in samples from B‐CLL patients and healthy adults were explored, by the six‐color flow cytometry technique. We focused on small T subpopulations, that is, DNT, DPT, and NKT‐like cells, given the recent findings about their promising antitumor ability. DNT cells have been shown to perform regulatory and immunoprotective functions.^7^ For the first time, Merims et al. demonstrated in acute myeloid leukemia (AML) patients that DNT lymphocytes can be expanded ex vivo even after intensive chemotherapy, and can kill both allogeneic and autologous primary leukemic blasts.^33^ Furthermore, they proved capable of bypassing the graft‐versus‐host disease, the main hurdle in cellular immunotherapy.^34^ Interestingly, DNT cells expanded under good manufacturing practice conditions, showed cytotoxic activity in myeloma, T‐cell leukemia, Burkitt lymphoma and AML in vitro and against AML in vivo without HLA restriction.^35^ Moreover, DNT cells proved to be a promising one‐therapy drug for cholangiocarcinoma as allogenic γδ T‐cell immunotherapy.^36^

The function of DPT cells is still controversial, with contrasting cytotoxic or suppressive activities for these cells.^12^ Bagot et al. studied the antitumor effect of DPTs in tumor growth, isolating the clone with CD4^+^ CD8dim+ phenotype from lymphocytes infiltrating a human major histocompatibility class II negative cutaneous T‐cell lymphoma.^37^ Desfrançois et al. observed an increased frequency of DPT cells in advanced breast cancer, suggesting this T‐cell subset as a potential regulator of immune responses to human breast cancer.^38^ Subsequently, it was reported an unprecedented significant increase in DPT frequency values, by multiparametric cytometry, in about 60% of melanoma tumors compared to blood samples.^39^ Furthermore, increased frequencies of reactive double positive (DP) infiltrate suggested a role in the metastatic process of colorectal carcinomas.^40^

NKT cells play a crucial function in cancer immunosurveillance and anti‐tumor immunity. Renukaradhya et al. published significant data regarding Type I NKT cells (iNKT) acting as anticancer effector cells, while Type II NKT (“non invariant” NKT) cells acting opposite their Type I counterparts.^41^ In particular, the Type II NKT cells seem to have a pro‐tumor role due to their capacity to increase tumor growth and enhance metastasis, whereas iNKT cells play an anti‐tumor role.^42^

A Phase II study in patients with head and neck squamous cell carcinoma evaluated anticancer effects and NKT cell‐specific immune responses in excised cancer tissue and PB, safety, and pathological effects.^43^ Using NKT‐deficient transgenic mouse models, Hix et al. demonstrated how Type I and Type II NKT cells regulate antitumor immunity of CD1d‐expressing breast tumors, pointing out the importance of evaluating tumor CD1d expression when customizing NKT‐based immunotherapies in metastatic breast cancer.^44^ Li et al. investigated NKT cell responses to B‐cell lymphoma, demonstrating that during lymphomagenesis, NKT cells control adaptive anti‐tumor immune response and significantly influence the disease outcome.^45^ A key role of NKT cells has also been highlighted in hematological malignancies.^46^, ^47^ A recent study supported the contribution of iNKT cells to CLL immune surveillance and highlighted iNKT cell frequency as a prognostic marker for disease progression.^48^ Jadidi‐Niaragh et al. suggest a protective role for NKT‐like cells in patients with CLL, which appears to be presumably downregulated by Treg cells.^49^ Blachnio et al. compared the number of T cells, including NKT‐like cells, in patients with CLL and healthy controls, during chemoimmunotherapy. The study concludes that the quantification of these subpopulations by flow cytometry during treatment may have prognostic significance.^50^

Very recently, our group performed a longitudinal monitoring of the RapidArc/moderate hypofractionation curative radiotherapy effects on the small subpopulations of T lymphocytes DNT, DPT, and NKT in patients treated for localized prostate cancer. DNT, DPT, and NKT cells significantly decreased at the end of RT with slight tendency to recover values during follow‐up, particularly in the hypofractionation group.^51^

A diagram depicting the previously observed roles of DNT, DPT, and NKT‐like cells is given in Figure S1.

As expected, in this study the percentages of each lymphocyte subpopulation evaluated showed a reduction from the values of healthy controls due to the increase in the percentage of CD19^+^ neoplastic B cells in CLL patients. However, through the comparison of all small T subpopulations stratified for their respective prognostic group versus healthy donors, an increase in DNT and NKT absolute values was found, while no significant changes were observed for DPT cells. The relevant variations highlighted with respect to controls may reflect an appropriate immune response to a malignant B‐cell clone, given their innate immunity.^3^, ^5^, ^6^, ^35^ The expansion of NKT‐like cells in Group A (that curiously did not significantly diminish as percentages) could be due to the significant expansion of WBC, contrary to NKT‐like cells in Groups B and C, that were significantly reduced with respect to control values as percentage value (Table S3). However, the same trend was found in the study of Bojarska‐Junak et al.^30^ in which the percentage of CD3^+^/CD16^+^ CD56^+^ cells was significantly decreased in patients who showed disease progression. Furthermore, the study showed that a significant correlation of absolute values of NKT‐like cells compared to B cells was observed only in prognostic Group B. This is probably due to a more uniform trend in Group B compared to Group A (which was slightly bigger but with more variable values). Prognostic Group C, with its small number, may be limited in comparison. No significant correlation of B cells with DNT or DPT cells (on total patients or by each prognostic group) was found. Thus, it is conceivable (at least for the prognostic Group B) that NKT‐like cells may intervene in the anti‐tumor response against B‐CLL cells.

These results could suggest that the monitoring of these cell numbers may provide helpful information for evaluating disease activity.

A significant increase in total T lymphocytes in patients with B‐CLL has previously been reported in the literature.^3^, ^4^, ^7^, ^23^, ^24^, ^31^ Based on previous data that reported the expansion of Tc and Th cells in B‐CLL,^52^, ^53^, ^54^, ^55^, ^56^, ^57^ we analyzed the contribution of all T subpopulations on T‐cell increase, focusing attention on DNT, DPT, and NKT‐like subsets. So, this study demonstrated that absolute CD3^+^ T‐cell counts were increased in B‐CLL patients largely due to the expansion of Tc, Th, and DNT, regardless of the prognosis group (Figure S5). In particular, best correlations were observed in Groups B and C, probably due to the staging. NKT‐like and DPT marginally caused the rise of absolute CD3^+^ T‐cell counts; in particular, only in Group B there was a positive correlation with the increase in T cells, not in groups A and C (Figures 4, 5, 6). The same statistical analysis was performed in healthy donors and, as expected, the correlations were higher for CD8 and CD4 cells, similar for DPT and NKT‐like cells, but curiously lower for DNT cells (Figure S6). The counterevidence in healthy donors and the significant correlation in patients with B‐CLL could suggest that the increase in DNT is due to an immune response of DNT cells to the disease. Analyzing the variations exclusively between prognostic groups, we found no significant variations for the subpopulations of interest.

These results supported the concept that active B‐CLL immune surveillance of DNT cells is independent of disease staging. In contrast, DPT and NKT‐like cells appear to increase significantly only as the disease progresses. These data must be confirmed by increasing the number of patients.

## 5 CONCLUSIONS

Considering recent findings about DNT, DPT, and NKT‐like cells, as potential therapeutic strategies and as key role in tumors, this retrospective study aimed to investigate the baseline values of these T subsets. For the first time, the values at diagnosis related to all three subpopulations of interest in patients with B‐CLL were evaluated. This led to a comparison of the obtained data between patients and healthy donors, between the prognostic groups identified and the T subpopulations of interest. The small number of patients belonging to the poor prognostic Group C probably did not provide clear and consistent results, placing some limitations on this study. We hypothesize, instead, that the initial staging of the disease for the prognostic Group A does not induce an active immune T response of NKT‐like and DPT cells. However, NKT‐like cell monitoring could be useful for evaluating disease progression. We can confirm recent scientific developments demonstrating the immune T nature of DNT cells.

These early observations are a very strong argument to encourage further studies on these minority T subpopulations by expanding patient casuistry. Specifically, the different trend of Tc, Th, NKT‐like, and DNT cells compared to DPT cells could be an interesting object of study to better understand their different functional participation in B‐CLL. In conclusion, our findings motivate us to conduct further studies aimed at elucidating the mechanisms underlying the increased accumulation of functional DNT, DPT, and NKT‐like cells in cancer, considering their role in the context of immunotherapies.

## AUTHOR CONTRIBUTIONS

**Luciana Valvano:** Conceptualization (equal); data curation (equal); investigation (equal); methodology (equal); writing – original draft (equal); writing – review and editing (equal). **Filomena Nozza:** Data curation (equal); investigation (equal); methodology (equal). **Giovanni D'Arena:** Data curation (supporting); formal analysis (supporting); writing – original draft (supporting); writing – review and editing (equal). **Fiorella D'Auria:** Investigation (equal); methodology (equal); writing – original draft (equal); writing – review and editing (equal). **Luciana De Luca:** Formal analysis (equal). **Giuseppe Pietrantuono:** Investigation (equal). **Giovanna Mansueto:** Investigation (supporting). **Oreste Villani:** Investigation (supporting). **Simona D'Agostino:** Investigation (supporting). **Daniela Lamorte:** Writing – original draft (supporting); writing – review and editing (equal). **Giovanni Calice:** Data curation (equal); investigation (equal); writing – original draft (supporting). **Teodora Statuto:** Data curation (supporting); investigation (equal); methodology (equal); writing – original draft (equal); writing – review and editing (equal).

## FUNDING INFORMATION

This work was supported by Italian Minister of Health ‐ Ricerca Corrente 2022.

## DISCLOSURE

The authors declare that they have no competing interests.

## ETHICS STATEMENT

This non‐interventional study was conducted according to the principles of the Declaration of Helsinki and approved by Comitato Etico Unico Regionale per la Basilicata (approval number 20200026759). Written informed consent was obtained from the participants for the publication of any potentially identifiable images or data included in this article.

## Supporting information


Figure S1.

Figure S2.

Figure S3.

Figure S4.

Figure S5.

Figure S6.

Table S1.

Captions

## Data Availability

Availability of data and materials The datasets used and/or analyzed during the current study are available from the corresponding author on reasonable request.
