# Supplementary figures and images for: Preliminary analysis of double‐negative T, double‐positive T, and natural killer T‐like cells in B‐cell chronic lymphocytic leukemia
Source: Cancer Med. 2023 May 4;12(12):13241–55. doi: 10.1002/cam4.6015 (PMC10315784; doi:10.1002/cam4.6015)

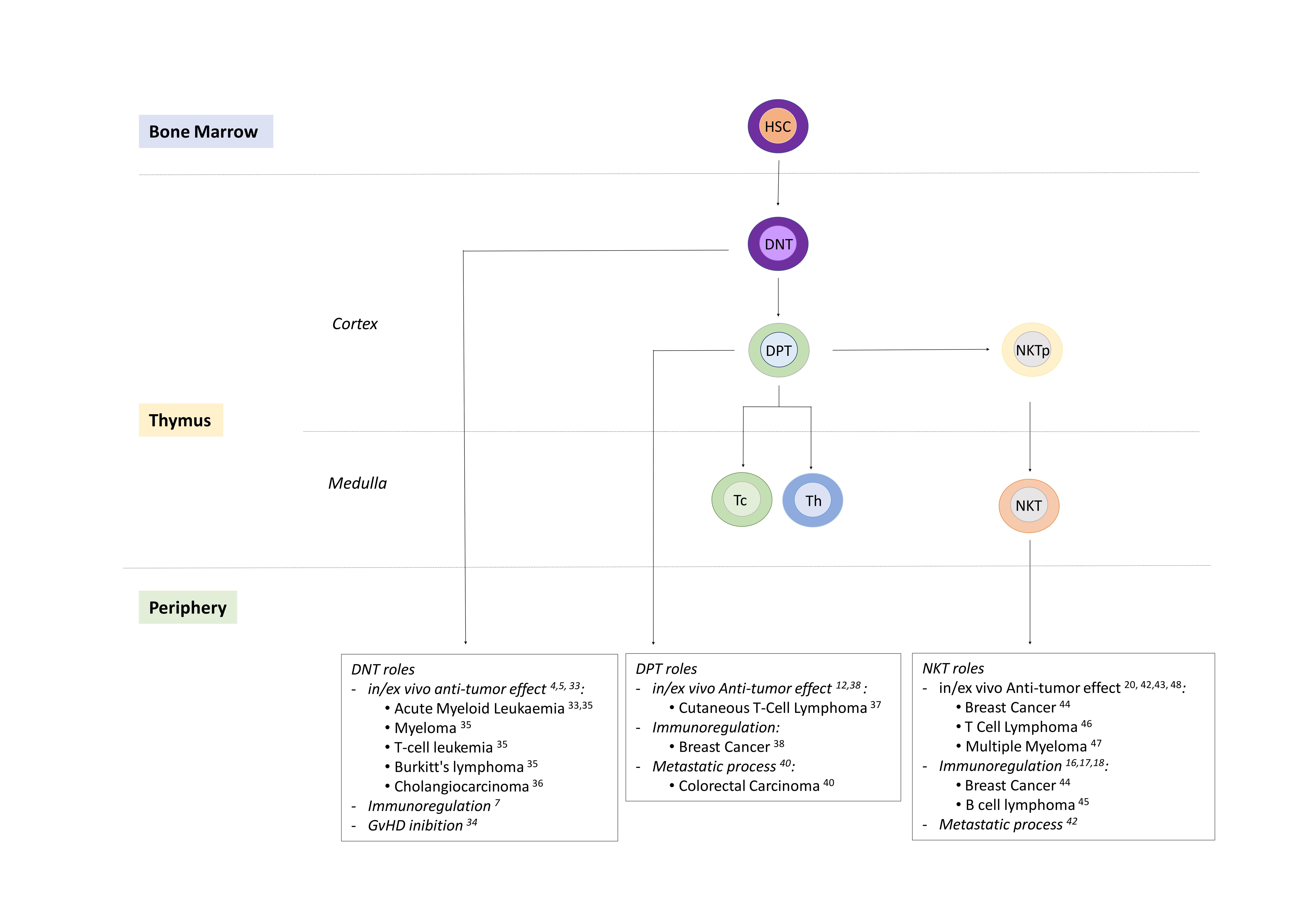

Supplement: Supplementary file 1 — Figure S1. [file CAM4-12-13241-s003.jpg]

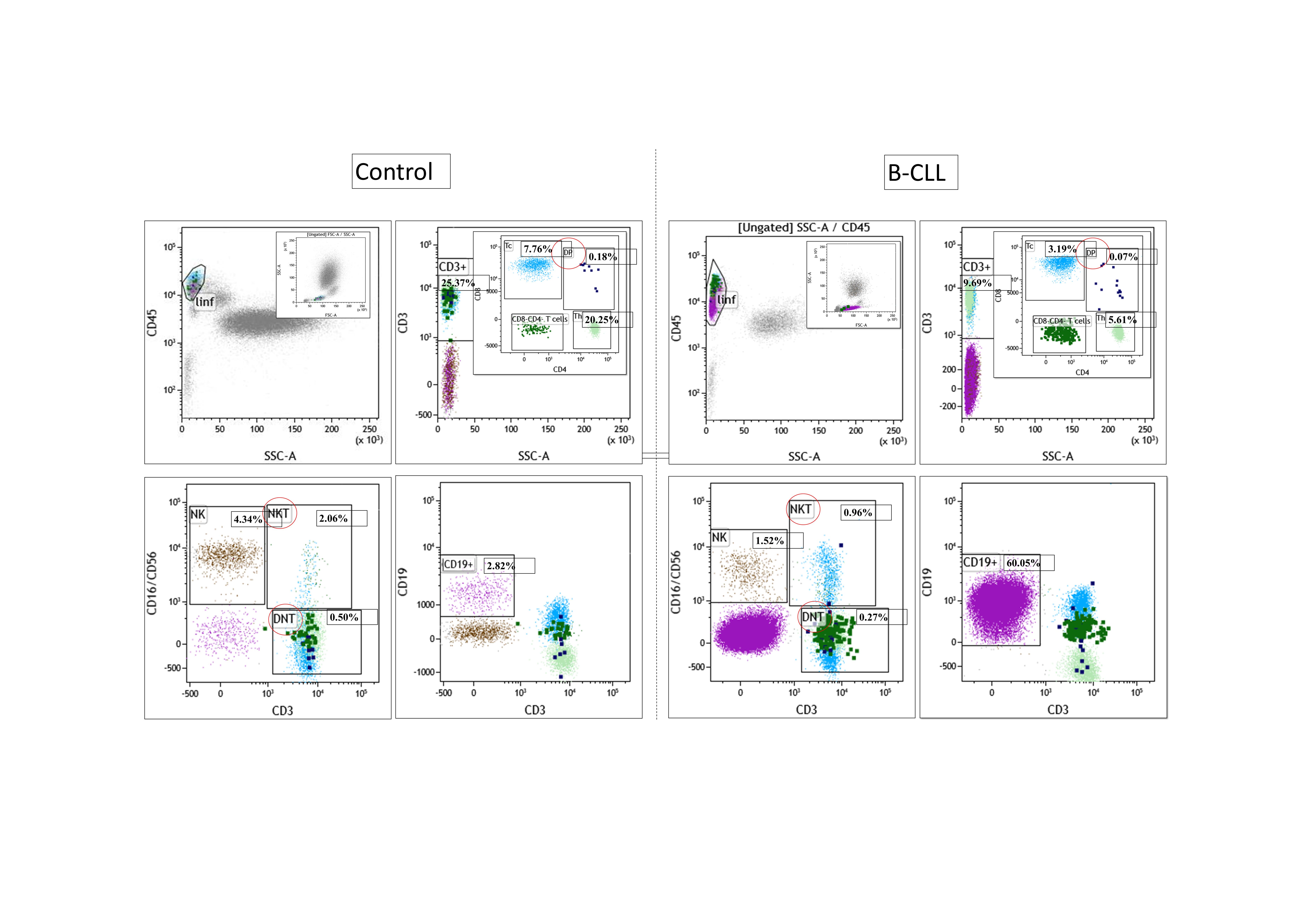

Supplement: Supplementary file 2 — Figure S2. [file CAM4-12-13241-s005.jpg]

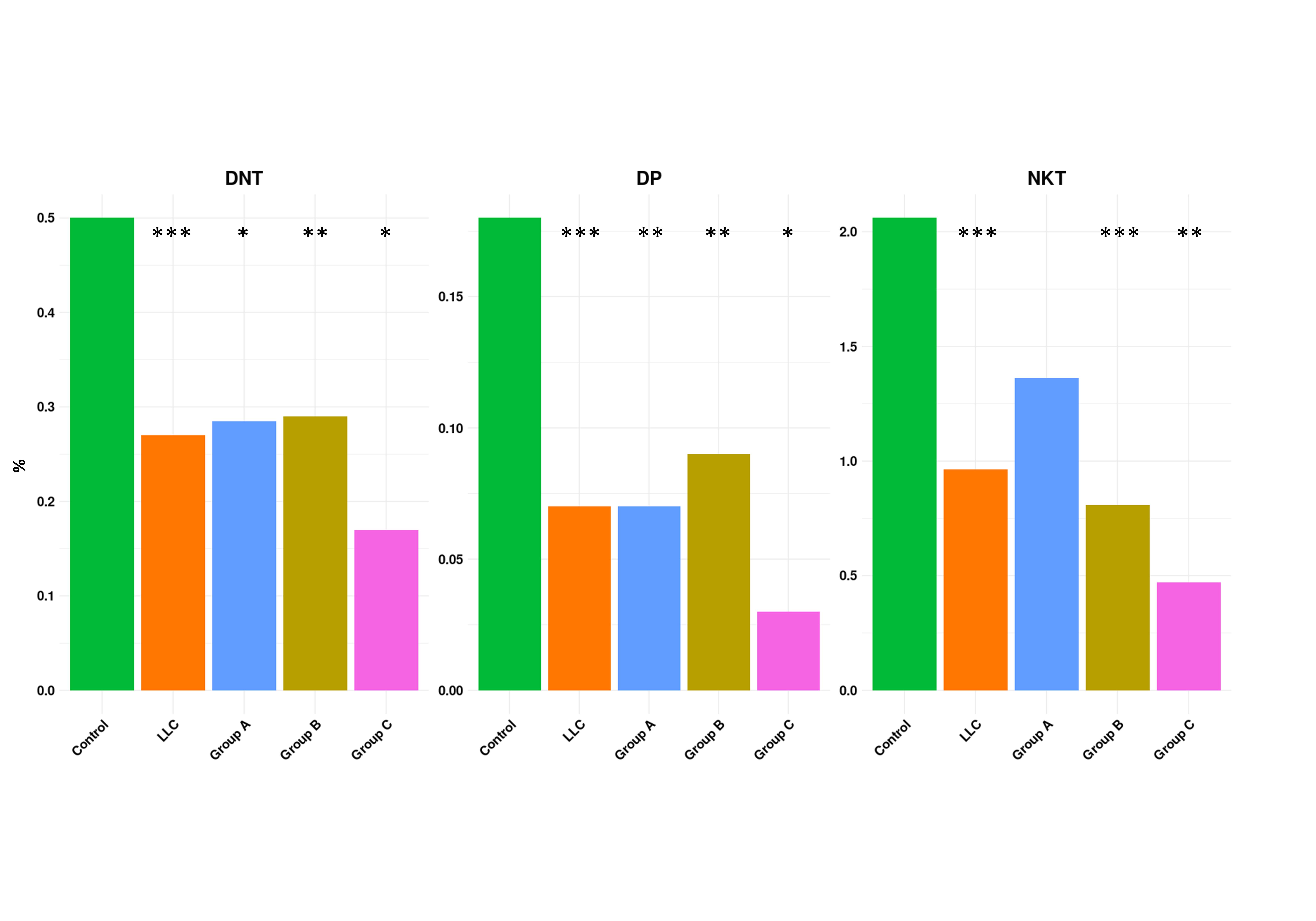

Supplement: Supplementary file 3 — Figure S3. [file CAM4-12-13241-s001.jpg]

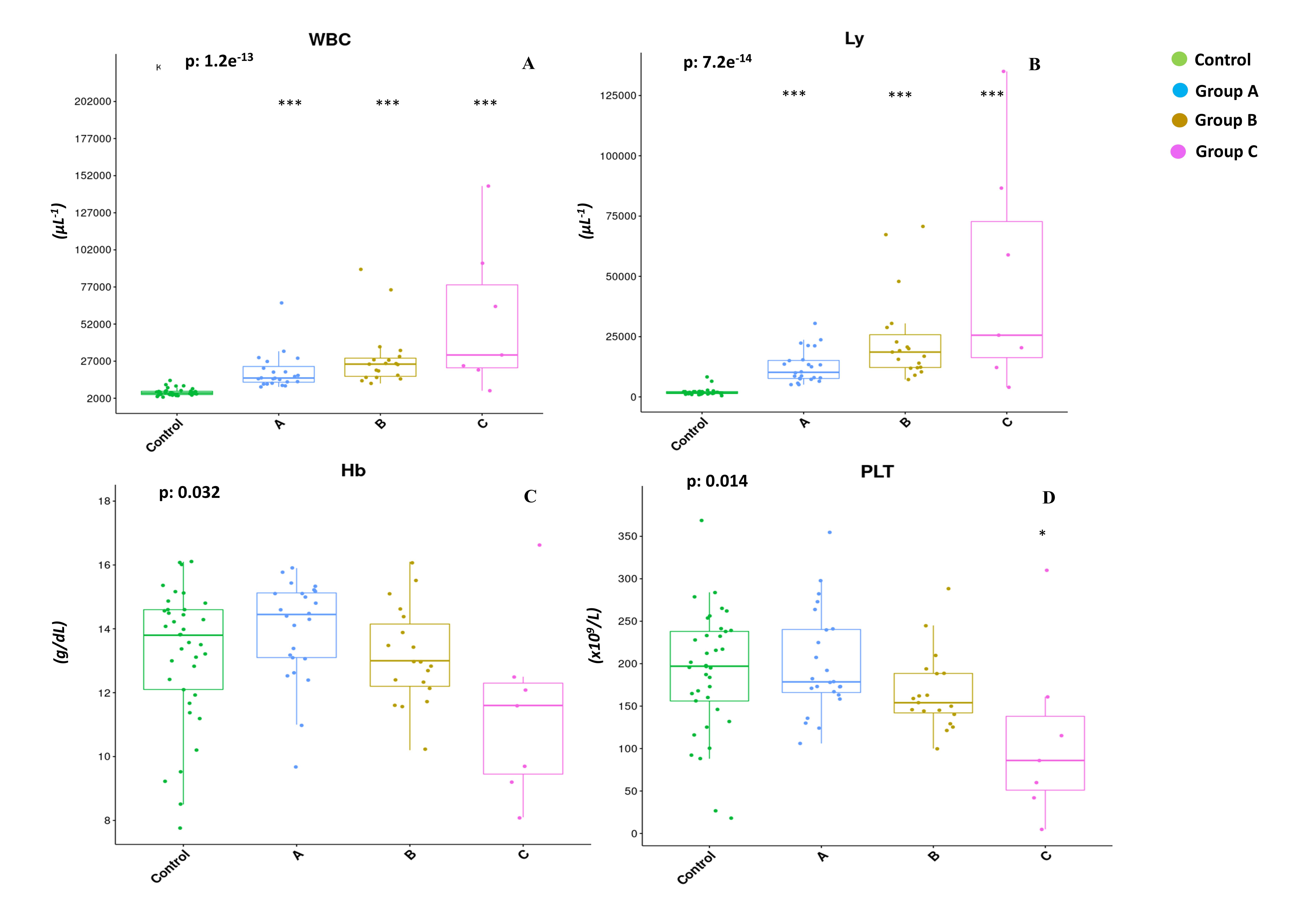

Supplement: Supplementary file 4 — Figure S4. [file CAM4-12-13241-s007.jpg]

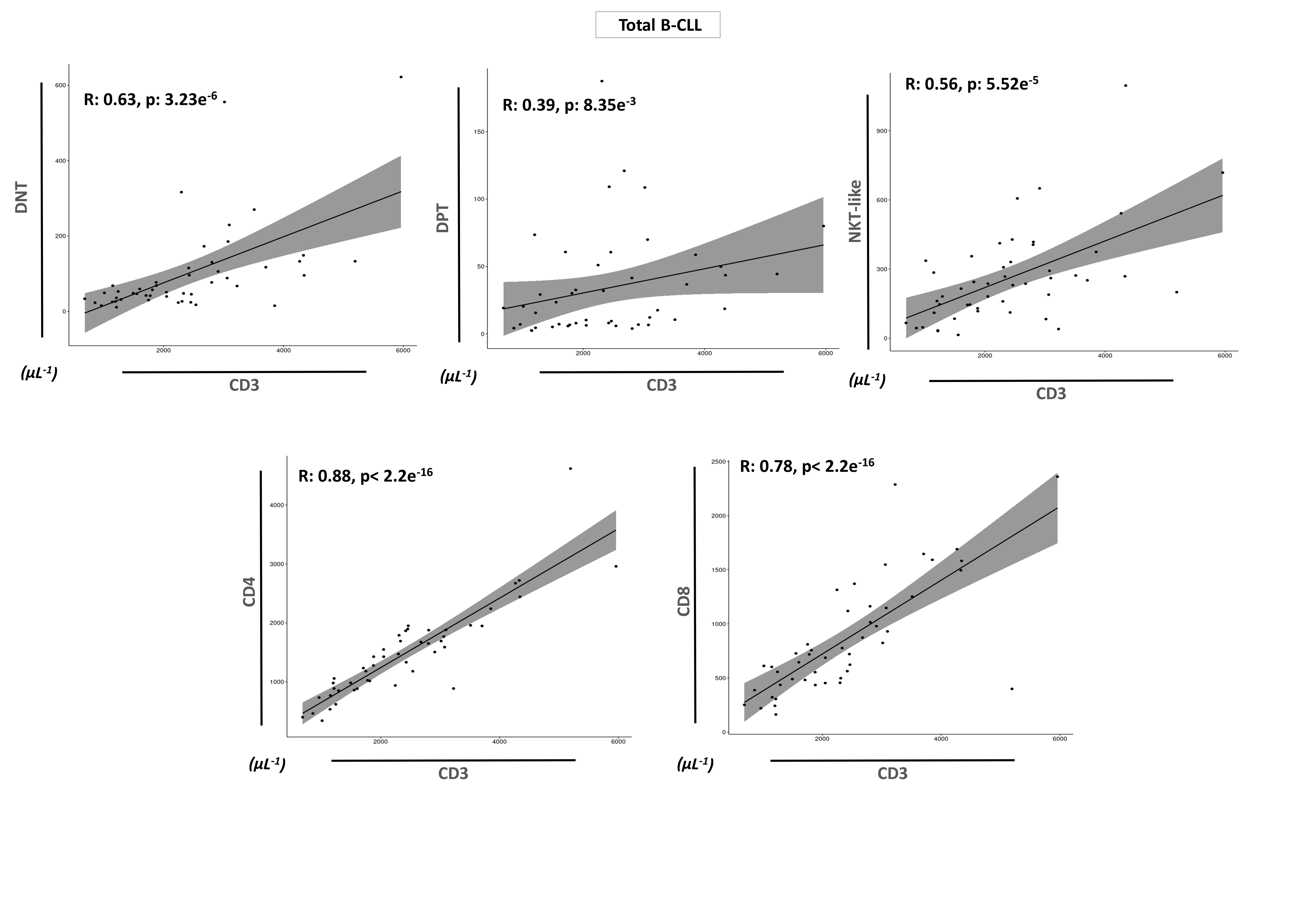

Supplement: Supplementary file 5 — Figure S5. [file CAM4-12-13241-s008.jpg]

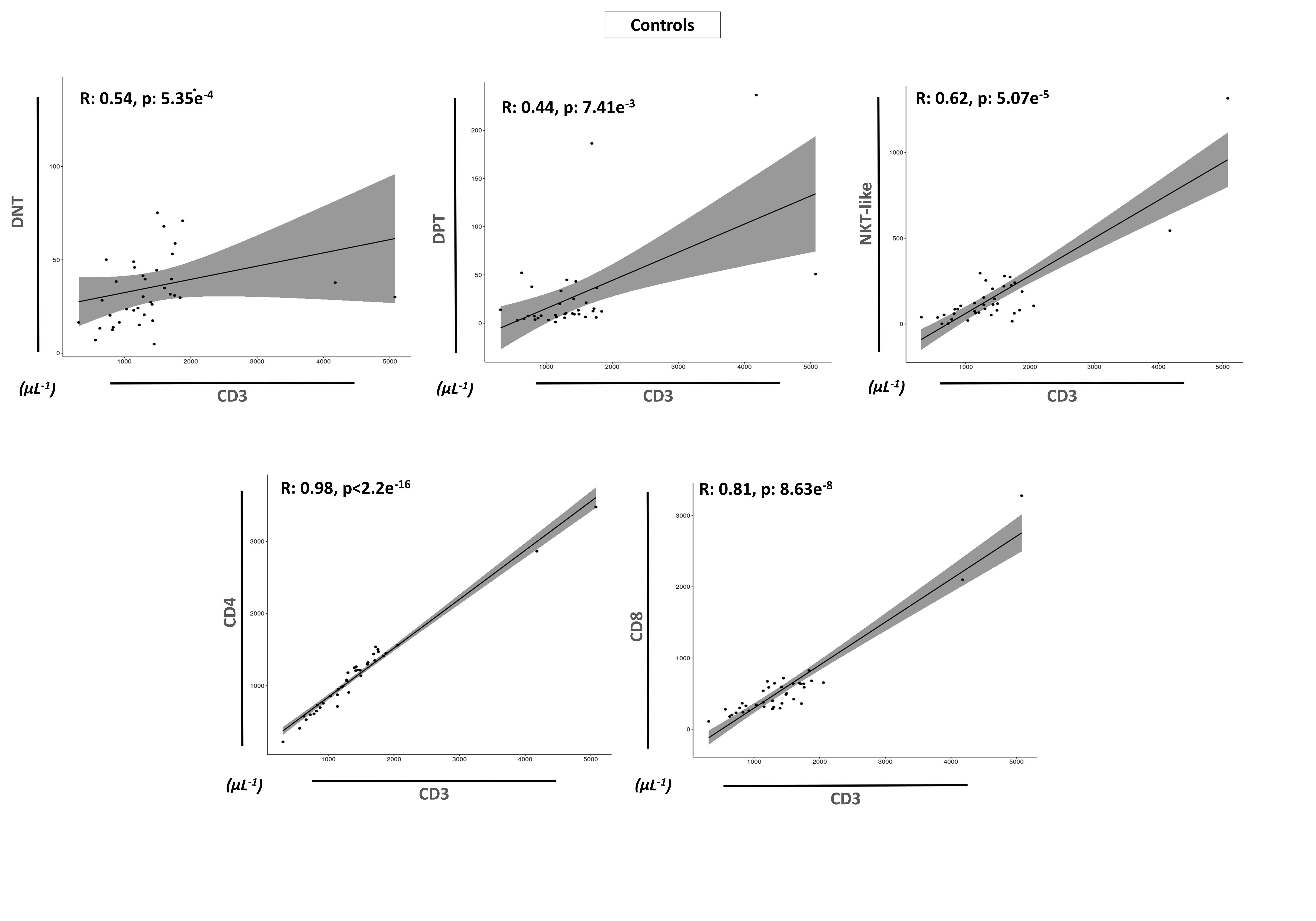

Supplement: Supplementary file 6 — Figure S6. [file CAM4-12-13241-s004.jpg]
