# Supplementary material for: Preliminary analysis of double‐negative T, double‐positive T, and natural killer T‐like cells in B‐cell chronic lymphocytic leukemia
Source: Cancer Med. 2023 May 4;12(12):13241–55. doi: 10.1002/cam4.6015 (PMC10315784; doi:10.1002/cam4.6015)
Supplement: Supplementary file 7 — Table S1. [file CAM4-12-13241-s002.docx]

*Supplementary Table 1.* A detailed list of panels used for flow cytometric analysis, including the antibody clone.
Flow cytometric analysis of lymphocyte subpopulations was performed with panels 1 or 2 or 3. CD38 expression on malignant cells (CD19 + CD5 +) was instead evaluated with panels 3 or 4. Monoclonal antibodies were purchased from BD or BC. Two samples was tested using BD's product "OneFlow™ LST Catalog No. 658619", not shown in the table.

| **Panel** | **FITC** | **PE** | **PerCP/PerCP-Cy™5.5** | **PE-Cy™7/PC7** | **APC** | **APC-Cy™7** |
| --- | --- | --- | --- | --- | --- | --- |
| **1  Internal laboratory combination of single antobodies  Clone  Brand** | CD3    SK7  BD | CD16 + CD56    B73.1 + NCAM16.2  BD | CD45    2D1  BD | CD8    SK1  BD | CD4    SJ25C1  BD | CD19    SJ25C1  BD |
| **2  Internal laboratory combination of single antobodies  Clone  Brand** | CD3    SK7  BD | CD16 + CD56    B73.1 + NCAM16.2  BD | CD4    SK3  BD | CD8    SK1  BD | CD19   SJ25C1  BD | CD45   2D1  BD |
| **3  Multitest 6-color TBNK Reagent  Catalog No: 662967   Clone  Brand** | CD3    SK7  BD | CD16 + CD56    B73.1 + NCAM16.2  BD | CD45    2D1  BD | CD4   SK3  BD | CD19   SJ25C1  BD | CD8   SK1  BD |
| **3  Internal laboratory combination of single antobodies  Clone  Brand** | CD5  BL1a   BC | CD49d  L25   BD | CD45   2D1   BD | CD38   LS198-4-3   BC | CD19  J3-119   BC | / |
| **4**  **Internal laboratory combination of single antobodies**  **Clone**  **Brand** | CD5    L17F12   BD | CD49d    L25   BD | CD45    2D1   BD | / | CD38    HB7   BD | CD19    SJ25C1  BD |

*Supplementary Table 2.* Distribution of lymphocyte subsets percentage values in the total B-CLL patients, in the different Rai-Binet stages and in the controls.   
Median and Interquartile Range (IQR) of percentages on total leukocytes (CD45+) are presented.  Asterisks indicate the result of the comparison between the percentage values of the various lymphocyte populations between CLL patients and healthy donors. p- value (evaluated by using Wilcoxon Rank test):  *
Significantly different from controls, P<0.05 **Significantly different from controls, P<0.01;  *** Significantly different from controls, P<0.001

| **Values** | B cells | T cells | NK cells | Tc cells | Th cells | DNT cells | DPT cells | NKT-like cells |  |
| --- | --- | --- | --- | --- | --- | --- | --- | --- | --- |
| **Control, n=38**  **Median % (IQR)** | 2.8  (2.4) | 25.4  (13.0) | 4.3  (3.0) | 7.8  (6.0) | 20.3  (8.7) | 0.5  (0.56) | 0.2  (0.4) | 2.1  (2.8) |  |
| **Total B-CLL n=50**  **Median % (IQR)** | 60  (34.5)  *** | 9.7  (9.2)  *** | 1.5  (1.3)  *** | 3.2  (5.8)  *** | 5.6  (6.6)  *** | 0.3  (0.4)  *** | 0.1  (0.2)  *** | 1  (1.1)  *** |  |
| **Group A, n=24**  **Median % (IQR)** | 46.6  (28.6)  *** | 14.1  (9.1)  *** | 1.7  (1.4)  *** | 4.5  (3.8)  *** | 9.3  (7.7)  *** | 0.3  (0.4)  * | 0.1  (0.2)  ** | 1.4  (1.1) |  |
| **Group B, n=19**  **Median % (IQR)** | 66.9  (25.3)  *** | 8.5  (5.4)  *** | 1.3  (1.0)  *** | 2.7  (3.4)  *** | 4.8  (3.4)  *** | 0.3  (0.4)  ** | 0.1  (0.2)  ** | 0.8  (0.8)  *** |  |
| **Group C, n=7 Median % (IQR)** | 79.5  (27.2)  *** | 4.7  (2.7)  *** | 1.0  (2)  *** | 1.8  (1.4)  *** | 2.7  (1.7)  *** | 0.2  (0.8)  * | 0  (0.2)  * | 0.5  (0.7)  ** |  |
|  | | | | | | | | | |

*Supplementary Table 3*. NKT percentages and absolute values. 
Median of absolute (µL^-1^) and percentages counts on total leukocytes (CD45+). p-value: **Significantly different from controls, P<0.01. *** Significantly different from controls, P<0.001.

|  | **Controls** | **Total C-LL** | **Group A** | **Group B** | **Group C** |
| --- | --- | --- | --- | --- | --- |
| **WBC (Median µL-1)** | 5490 | 20675  *** | 15630  *** | 25000  *** | 31160  *** |
| **NKT-like cells (%)** | 2.06 | 0.96  *** | 1.36 | 0.81  *** | 0.47  ** |
| **NKT-like cells (Median µL-1)** | 97.24 | 233,45  *** | 245.52  *** | 236.73 | 147.05 |
|  | | | | | |
