# Supplementary material for: Preliminary analysis of double‐negative T, double‐positive T, and natural killer T‐like cells in B‐cell chronic lymphocytic leukemia
Source: Cancer Med. 2023 May 4;12(12):13241–55. doi: 10.1002/cam4.6015 (PMC10315784; doi:10.1002/cam4.6015)
Supplement: Supplementary file 8 — Captions [file CAM4-12-13241-s006.docx]

*Supplementary Figure 1*. DNT, DPT, and NKT cells development and roles. ​
The migration of hematopoietic stem cells (HSC) from bone marrow to thymus marks the beginning of the process of T-cell development. In the thymic cortex, Double Negative T (DNT) cells gradually differentiate and in the last stage of development DNT are converted into Double Positive T (DPT) cells. Only a few DPT cells are selected and assigned to the Natural Killer T (NKT) precursor (NKTp). Finally, mature NKT and single positive T cells (Cytotoxic T and Helper T cells, Tc and Th) pass from medulla to peripheral circulation.


*Supplementary Figure 2*. A general picture of the gates analyzed and the differences highlighted between CLLs and healthy donors. Median percentages are evaluated on CD45+ cells. 

*Supplementary Figure 3*. Histogram distribution of percentage values of DNT, DPT and NKT-like cells.
The histogram shows the difference between the percentage values ​​of DNT, DPT and NKT-like cells in the total of patients affected by B-cell chronic lymphocytic leukemia (B-CLL), in each prognostic group and in controls.
p-value (evaluated by using Wilcoxon Rank test): Significantly different from controls * P<0.05; 
** P<0.01; *** P<0.001. 


*Supplementary Figure 4*. The impact of RAI-BINET staging in the absolute counts of WBC (panel A), Ly (panel B), Hb (panel C) and PLT (panel D) respect to controls.  
Each symbol represents single subject and vertical bars represent median values. Statistically significant analyses (respect the healthy donors) are indicated by asterisks: *p < 0.05, **p < 0.01 and ***p < 0.001.   
p-value reported in the box plots panel refers to Kruskal-Wallis test. 

*Supplementary Figure 5*. Relationship between DNT, DPT and NKT-like cells and the increase in CD3 cells in B-CLL patients.
Spearman’s correlation coefficient (R) test between the absolute values of CD3+ cells (µl^-1^) and all T-cells subsets (µl^-1^) (DNT, DPT, NKT, CD8 and CD4) in total B-CLL patients. Statistical analysis is indicated with the p-value.

Supplementary Figure 6. Relationship between DNT, DPT and NKT-like cells and the increase in CD3 cells in healthy donors.
Spearman’s correlation coefficient (R) analysis between the absolute values of CD3+ cells (µl^-1^) and all T-cells subsets (µl^-1^) (DNT, DPT, NKT, CD8 and CD4) in controls. Statistical analysis is indicated with the p-value.
